# Association between triglyceride glucose index and hyperuricemia: a new evidence from China and the United States

**DOI:** 10.3389/fendo.2024.1403858

**Published:** 2024-07-01

**Authors:** Ruoyu Gou, Danni Dou, Mi Tian, Xiaoyu Chang, Yonggang Zhao, Xin Meng, Guanghua Li

**Affiliations:** ^1^ School of Public Health, Ningxia Medical University, Yinchuan, Ningxia, China; ^2^ School of Basic Medicine, Ningxia Medical University, Yinchuan, Ningxia, China

**Keywords:** TyG, hyperuricemia, NHANES, CHARLS, HUA

## Abstract

**Background:**

Hyperuricemia (HUA) is a glo\bal public health problem. The etiology of HUA is complex and efficient and accurate assessment metrics are still lacking when conducting large-scale epidemiologic screening. The aim of this study was to evaluate the association of the triglyceride glucose (TyG) index, TyG-body mass index (BMI), TyG-waist-to-height ratio (WHtR) with the risk of HUA.

**Methods:**

Based on data collected from the National Health and Nutrition Examination Survey (NHANES) in the United States and the China Health and Aging Longitudinal Study (CHARLS) in China, a total of 14,286 U.S. adults and 4,620 Chinese adults were included in the analysis. The study examined the levels of TyG, TyG-BMI, TyG-WHtR, and TyG-WC. Multivariate logistic regression was utilized to investigate the relationships between these variables and hyperuricemia (HUA), separately. Additionally, the study used restricted cubic splines (RCS) to explore the linear associations of TyG, TyG-BMI, TyG-WHtR, TyG-WC, and HUA, separately.

**Results:**

The NHANES results showed that TyG [Q2, 1.58(1.26, 1.98); Q3, 2.36 (1.94, 2.88); Q4, 3.21 (2.61, 3.94)], TyG-BMI [Q2, 2.14 (1.74, 2.65); Q3, 3.38 (2.74, 4.17); Q4, 6.70 (5.55, 8.02)], TyG-WHtR [Q2, 1.92 (1.56, 2.36); Q3, 3.14 (2.56, 3.85); Q4, 6.28 (5.12, 7.69)], TyG-WC [Q2, 2.32 (1.85, 2.90); Q3, 3.51 (2.84, 4.34); Q4, 7.32 (5.95, 9.02)] were identified as risk factors for hyperuricemia (HUA). Similarly, the CHARLS results, when fully adjusted for covariates, indicated that TyG [Q4, 2.36 (1.08, 5.15)], TyG-BMI [Q3, 2.60 (1.05, 6.41); Q4, 3.70 (1.64, 8.32)], TyG-WHtR (Q4, 2.84 (1.23, 6.55), TyG-WC [Q4, 2.85 (1.23, 6.5)] were also risk factors for HUA. The predictive ability of each indicator for the risk of developing HUA was stronger in women than in men. Furthermore, there was an observed nonlinear relationship between TyG, TyG-BMI, TyG-WHtR, TyG-WC, and HUA in both the NHANES and CHARLS datasets (*P-nonlinearity* < 0.05).

**Conclusion:**

These findings suggest that TyG, TyG-BMI, TyG-WHtR and TyG-WC are associated with an increased risk of HUA. They are potential indicators for screening HUA status in the general population in China and the United States.

## 1 Introduction

Hyperuricemia (HUA) is characterized by elevated serum uric acid (SUA) levels, often attributed to either heightened uric acid synthesis or diminished urinary elimination. Uric acid, a byproduct of purine breakdown, is primarily eliminated via the kidneys, as humans lack the enzyme uricase. Disruptions in purine metabolism, increased uric acid production, or impaired excretion can result in uric acid dysregulation, leading to various complications like gout, kidney stones, metabolic syndrome, hypertension, and cardiovascular disease (1–3), the etiology of HUA has not been fully elucidated. The prevalence of HUA is increasing with the continuous improvement of living standards and changes in people’s dietary habits (4). HUA has been reported in 14.0% of Chinese adults in a 2018 survey (5), and according to the NHANES, approximately 20% of U.S. adults have HUA (6), and it is estimated that approximately 8.9-24.4% of the general population have HUA (7). In recent years, HUA has emerged as a significant global public health concern.

Insulin-normoglycemic clamp is the gold standard for assessing IR status (8). The assessment process for IR is invasive, expensive, and complex, making it less than ideal for routine clinical monitoring. However, there are other practical and feasible tools available that do not rely on serum insulin levels to assess IR status (9). Some novel and simple metrics, such as TyG, TyG-BMI, TyG-WHtR and TyG-WC, and TyG-waist circumference (WC), have been reported as reliable alternatives for assessing IR (10–12). When TyG is combined with other indicators of obesity such as BMI, WC, and WHtR, its effectiveness in assessing IR may be enhanced (13, 14), may be more feasible and practical than other expensive methods. The TyG index demonstrates a high sensitivity of 96.5% and specificity of 85.0% in diagnosing IR when compared to IR (15). This suggests that TyG-associated markers in combination with the obesity index are more effective as surrogate markers than TyG in reflecting the degree of IR (9, 16). A Chinese study pointed out that high levels of TyG-WC are an independent risk factor for First Myocardial Infarction in patients with hypertension and OSA (17) and are associated with an increased risk of CKD (18). Additionally, TyG predicted mortality risk and was synergistic with SUA on mortality risk (19). It is well known that the lower the glomerular filtration rate (GFR), the higher the prevalence of hyperuricemia and gout (20). However, for patients at cardiovascular risk, hyperuricemia is a risk factor for cardiovascular and all-cause mortality in addition to eGFR and proteinuria (21).

Several studies have found a higher prevalence of HUA in diabetic patients compared to non-diabetic individuals. The strong association between HUA and the severity of diabetes mellitus indicates a potential relationship with blood glucose levels (6). Previous studies have shown that insulin resistance (IR) may contribute to HUA (22) and that reducing IR may reduce SUA levels and subsequent risk of gout (23, 24). TyG-derived indices seem to correlate more strongly with the risk of hyperuricemia than TyG indices (15). Additionally, a Mendelian randomization study indicated that pancreatic hyperinsulinemia may induce HUA, Zhu et al. showed that elevated UA levels preceded IR (25), and Hu et al. revealed a correlation between elevated UA levels and elevated risk of IR by Mendelian randomization analysis, but no significant causal relationship was found (26). A study from Jiangxi Province, China, reported that an increase in IR indices was associated with the risk of HUA in hypertensive patients (27). Another study noted that the effect of TyG index on HUA in hypertensive patients was closely related to the degree of hypertension prevalence, and the correlation between TyG index and HUA was significantly higher in patients with grade 1-2 hypertension than in patients with grade 3 hypertension (22). The findings of another study are more clinically relevant, the degree of association between hyperuricemia and lipids varied when different cutoff values were used to diagnose hyperuricemia, in the Uric Acid Right for Heart Health (URRAH) study (28). The study indicated that the diagnostic criteria for uric acidemia was 6.0 mg/dl for women and 7.0 mg/dl for men, but the new finding in the URRAH study was 5.1 mg/dl for women and 5.6 mg/dl for the form (28).

Current research on the relationship between TyG index and SUA levels is limited, with most studies focusing on Chinese populations. This study, however, provides a nationally representative analysis of the impact of four IR substitutes on HUA development in both China and the United States. By utilizing data from CHARLS and NHANES, which offer broad geographic coverage and rigorous implementation procedures, our findings are reflective of the general population in both countries. While previous studies have supported this hypothesis, they often lack generalizability. The hypothesis is that TyG and its derived indices are risk factors for HUA and can increase the risk of developing HUA. Early detection and management of IR in HUA patients, prior to the onset of clinical symptoms, could aid in managing HUA and preventing IR-related comorbidities, with significant insights provided by individual studies.

## 2 Materials and methods

### 2.1 Data sources and study population

**Figure 1:**
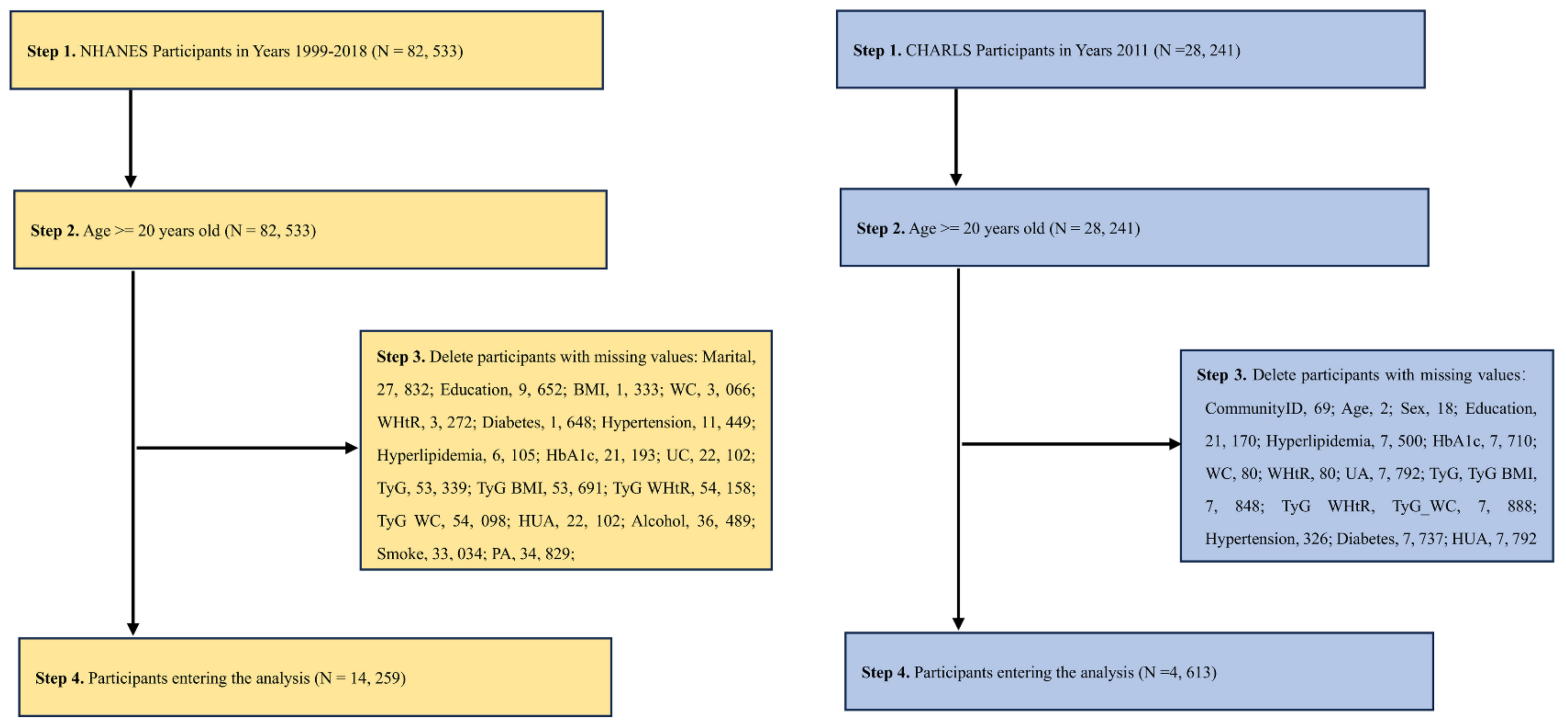
A flow chart of the NHANES [**(A)** 1999-2018, Year cycle] and CHARLS [**(B)** 2011-2012 survey data]. The strategy of extracting the variables and then directly calculating the required indicators and then removing the missing values was used.

Two large nationally representative datasets from China [China Health and Aging Longitudinal Study (CHARLS)] and the United States [National Health and Nutrition Examination Survey (NHANES)] were used. For China, we use CHARLS 2011-2012 survey data, and for the U.S., we aggregate data from 10 NHANES cycles from 1999-2018.The details of CHARLS and NHANES have been described elsewhere (29, 30). CHARLS is a nationally representative survey being conducted in China. NHANES is a nationally representative cross-sectional survey of the noninstitutionalized U.S. population. Using stratified multistage probability sampling methods, data were collected in two-year cycles beginning in 1999-2000, selecting a series of nationally representative samples of noninstitutionalized U.S. adults. The NHANES program was approved by the Ethics Review Board of the National Center for Health Statistics. Both CHARLS and NHANES collect information on demographic characteristics, medical history, prescription drug use, and laboratory tests. Results from CHARLS and NHANES can be weighted to obtain nationally representative estimates. CHARLS (http://charls.pku.edu.cn/) and NHANES (http://www.cdc.go/nchs/nhanes.htm) data are available on their respective Web sites All participants provided written informed consent. The participant screening process is illustrated in **Figure 1**.

### 2.2 Definitions of TyG and TyG related indices

The indices in the study were defined as follows: WHtR is defined as WC divided by body ­height (31); TyG = ln [fasting triglyceride (mg/dL) × fasting glucose (mg/dL)/2] (11); TyG-BMI = TyG × ­ BMI (12); TyG-WHtR = TyG × ­ WHtR (9); TyG-WC = TyG × ­ WC (12). TyG, TyG-BMI, TyG-WHtR, and TyG-WC were calculated as continuous variables.

### 2.3 Assessment of lifestyle factors

CHARLS participant Lifestyle Assessment: Lifestyle scores were derived from five factors: smoking, alcohol consumption, social activities, sleep duration, and BMI. Each factor was assigned a score of either 1 for yes or 0 for no. Smoking status was scored as 1 if the participant had never smoked, otherwise it was scored as 0. Similarly, drinking status was scored as 1 if the participant had not consumed alcohol in the past year, otherwise it was scored as 0. Participation in social activities was scored as 1 if the participant engaged in any social activities in the last month, otherwise it was scored as 0. Participants provided yes (1) or no (0) responses for each factor. A sleep duration score of 1 was given if the participant slept more than 6 hours on a typical night, otherwise it was scored as 0. Height and weight measurements were taken using standardized methods, and BMI was calculated as kg/m2. For participants not classified as underweight (BMI ≥18.5 kg/m2), the BMI score was set to 1, otherwise it was set to 0. The composite lifestyle score, ranging from 0 to 5, was calculated by combining the scores of these five factors. We then applied weights to the composite lifestyle scores (32).

NHANES participant Lifestyle Assessment: Because multiple lifestyle factors are interrelated and associated with HUA, we developed a Healthy Lifestyle Score based on a previous NHANES study. This score took into account smoking status, alcohol consumption, physical activity levels, and diet quality. Non-smoking was considered indicative of a healthy lifestyle. Healthy alcohol consumption was defined as one drink or less per day for women and two drinks or less per day for men, in line with the U.S. Dietary Guidelines. Physical activity was measured in metabolic equivalent hours per week, with the top third of participants classified as having a healthy level of physical activity. Dietary quality was evaluated using Healthy Eating Index (HEI) scores. The HEI-2015 is based on the 1999-2014 survey cycle and is consistent with the 2015-2020 Dietary Guidelines for Americans. Healthy eating was defined as a HEI in the top two quintiles of the distribution. For each lifestyle factor, a score of 1 was assigned to healthy levels and 0 to unhealthy levels. Thus, the Healthy Lifestyle Score is a sum of scores ranging from 0 to 5, with higher scores indicating healthier lifestyles. Although this simple cumulative approach has been widely used, with the underlying assumption that the associations between different lifestyle factors and outcomes are the same, this may not be correct. We therefore constructed a weighted lifestyle score in which each lifestyle factor is weighted according to its association with the outcome (33).

### 2.4 Assessment of HUA and other covariates

According to a previous study, HUA was defined as SUA ≥ 7.0 mg/dL in men and ≥6.0 mg/dL in women (34, 35). Serum uric acid values (continuous variables) were transformed into dichotomous variables (HUA/Non - HUA). Demographic information was collected by questionnaire, including age, sex, marital status (Married, Separated, Never married), Education (Primary school, High school or above), Healthy lifestyle score(It has been described in detail). The diagnosis of diabetes consists of the following components, any of which can be met to be diagnosed with diabetes: (1) the individual has been diagnosed with diabetes by a doctor; (2) glycosylated hemoglobin (HbA1c) (%) > 6.5; (3) fasting blood glucose (DM, mmol/l) ≥ 7.0; (4) random blood glucose (mmol/L) ≥ 11.1 and (5) use of diabetes medication or insulin. Hyperlipidemia was diagnosed by meeting any of the following criteria: hypertriglyceridemia (TG ≥ 150 mg/dL) and/or hypercholesterolemia (TC 240 mg/dL, LDL 160 mg/dL, HDL <40 mg/dL. Definition of hypertension: 1) taking hypertensive medication; 2) being informed by a licensed physician of hypertension or stated in the questionnaire to take prescribed medication for hypertension; 3) measuring the participant’s systolic blood pressure (SBP) mean ≥ 140 mmHg and/or diastolic blood pressure (DBP) ≥ 90 mmHg (mean of three times). Any one of the above three conditions can be diagnosed as hypertension. Since ethnicity information was not published in the CHARLS (2011, years) survey, the ethnicity variable was not adjusted in NHANES for consistency.

### 2.5 Statistical analysis

All remaining statistical analyses were performed using R software (4.2.2, https://cran.r-project.org/bin/windows/base/old/4.2.2/), with statistical tests being two-sided and considered statistically significant when the *P-value* < 0.05. To obtain statistics representative of U.S. adults, we utilized oversampling, stratification, and clustering techniques in the NHANES and CHARLS analyses. Weight-adjusted statistical tests were thoroughly taken into account. Demographic characteristics of participants’ HUA status were assessed using the chi-square test and t-test. Continuous variables indicators TyG, TyG-BMI, TyG-WHtR, and TyG-WC were grouped into quartiles. The continuous variables were divided into four groups and transformed into categorical variables. Multivariable logistic regression models were used to estimate the P - value, odds ratio (OR), and 95% confidence interval (CI) between TyG, TyG-BMI, TyG-WHtR, and TyG-WC and the risk of HUA, separately. Additionally, a gender-stratified analysis was conducted using multifactor logistic regression. (Model 1, No adjustment for any potential influence factors; Model 2, Adjusted for Sex, Age; Model 3, Adjusted for Sex, Age, Marital, Education levels and Healthy Lifestyle Score). Restricted Cubic Spline (RCS) plots were used to show trends in the partially significant variables of the multivariate logistic regression, separately. The exposure factors (TyG, TyG-BMI, TyG-WHtR, and TyG-WC) were entered into the RCS model as continuous variables. The RCS was used to test for nonlinear associations between exposure factors (TyG, TyG-BMI, TyG-WHtR, TyG-WC) and HUA, separately. (Adjusted for Sex, Age, Marital, Education levels and Healthy Lifestyle Score). Additionally, we excluded those with hypertension, dyslipidemia, and diabetes, and conducted sensitivity analyses using multivariate logistic regression models to assess the stability of the results, separately (adjusted covariates were consistent with the main analysis).

## 3 Results

### 3.1 Baseline characteristics

**Table 1-1** reports the sociodemographic characteristics of NHANES, a study with a total of 14,259 NHANES participants (Males, 52.67%; Females, 47.33%) We found that the mean age of the participants involved in the study was 45.92 years and the mean age of the participants with HUA was 48.44 years (Male, 63.10%; Female, 36.90%). In patients with HUA, TyG, TyG-BMI, TyG-WHtR and TyG-WC were higher than in Non-HUA participants (*P-value* < 0.001). Sex, Education, Marital, Diabetes, Hypertension, Hyperlipidemia, Healthy Lifestyle Score, WC, HbA1c, UA, WHtR and BMI were lower in participants with HUA compared to Non-HUA participants, which were significantly different (*P-value* < 0.05). Significantly, participants with HUA had lower Healthy Lifestyle Scores (*P-value* < 0.001). The sociodemographic characteristics of CHARLS are reported in **Table 1-2**. The total number of CHARLS participants in this study was 4,613 (males, 30.09%; females, 69.91%) We found that the mean age of the participants who participated in the study was 68.52 years, and the mean age of the participants with HUA was 71.65 years (Male, 73.27%; Female, 26.73%). In patients with HUA, TyG, TyG-BMI, TyG-WHtR, TyG-WC, UA and BMI were higher than in Non-HUA participants (*P-value* < 0.05). Education, Marital, Diabetes, Hyperlipidemia were healthy levels lower in participants with HUA compared to Non-HUA participants, with statistically significant differences (*P-value* < 0.05).

**Table 1-1:** Socio-demographic characteristics of US adults with HUA by NHANES survey cycle, 1999-2018.

Parameters	No. of Participants (Weighted %)			
	Total (N = 14, 259)	Non-HUA (N = 11, 376)	HUA (N = 2, 883)	*P-value*
Age	45.92 (0.25)	45.31 (0.27)	48.44 (0.39)	< 0.001
TyG	8.60 (0.01)	8.53 (0.01)	8.88 (0.02)	< 0.001
TyG BMI	245.39 (0.87)	236.04 (0.86)	284.13 (1.67)	< 0.001
TyG WHtR	4.97 (0.01)	4.82 (0.01)	5.58 (0.03)	< 0.001
TyG WC	843.78 (2.52)	816.81 (2.42)	955.43 (4.56)	< 0.001
WC	97.67 (0.24)	95.34 (0.24)	107.33 (0.41)	< 0.001
HbA1c, mmol/mol	5.52 (0.01)	5.50 (0.01)	5.63 (0.02)	< 0.001
UC	5.48 (0.02)	5.01 (0.01)	7.42 (0.02)	< 0.001
WHtR	0.58 (0.00)	0.56 (0.00)	0.63 (0.00)	< 0.001
BMI	28.40 (0.09)	27.54 (0.09)	31.93 (0.17)	< 0.001
**Sex**				< 0.001
Female	6623 (47.33)	5512 (49.85)	1111 (36.90)	
Male	7636 (52.67)	5864 (50.15)	1772 (63.10)	
**Education**				0.01
Primary school	3046 (13.63)	2486 (13.97)	560 (12.22)	
High school or above	11213 (86.37)	8890 (86.03)	2323 (87.78)	
**Marital**				0.02
Married	8878 (66.11)	7105 (66.31)	1773 (65.32)	
Separated	2757 (16.03)	2116 (15.56)	641 (18.00)	
Never married	2624 (17.85)	2155 (18.13)	469 (16.68)	
**Diabetes**				< 0.001
No	1256 (6.42)	898 (5.62)	358 (9.73)	
Yes	13003 (93.58)	10478 (94.38)	2525 (90.27)	
**Hypertension**				< 0.001
No	8679 (65.67)	7422 (69.86)	1257 (48.33)	
Yes	5580 (34.33)	3954 (30.14)	1626 (51.67)	
**Hyperlipidemia**				< 0.001
No	4052 (29.44)	3569 (32.26)	483 (17.79)	
Yes	10207 (70.56)	7807 (67.74)	2400 (82.21)	
**Healthy Lifestyle Score**				< 0.001
0	497 (3.83)	365 (3.46)	132 (5.40)	
1	2578 (18.33)	1917 (17.19)	661 (23.01)	
2	4923 (34.25)	3839 (33.43)	1084 (37.68)	
3	4180 (28.81)	3435 (29.64)	745 (25.35)	
4	1803 (12.63)	1577 (13.96)	226 (7.15)	
5	278 (2.15)	243 (2.33)	35 (1.40)	

Percentages were adjusted for NHANES survey weights. The P-value was calculated using a chi-square test and Students T test after considering the sampling weights. P-value <0.05.

**Table 1-2:** Socio-demographic characteristics of China adults with HUA by CHARLS survey cycle, 2011-2012.

Parameters	No. of Participants (Weighted %)			
	Total (N = 4, 613)	Non-HUA (N = 4, 273)	HUA (N = 340)	*P-value*
Age	68.52 (0.43)	68.28 (0.44)	71.65 (1.44)	0.020
TyG	8.69 (0.03)	8.66 (0.03)	9.05 (0.09)	< 0.001
TyG BMI	198.32 (1.51)	196.85 (1.55)	217.56 (4.97)	< 0.001
TyG WHtR	4.77 (0.03)	4.73 (0.03)	5.21 (0.16)	0.004
TyG WC	731.08 (5.20)	726.73 (5.16)	787.87 (22.51)	0.010
WC	83.97 (0.54)	83.74 (0.55)	86.98 (2.23)	0.160
HbA1c, mmol/mol	5.26 (0.03)	5.25 (0.03)	5.39 (0.08)	0.140
UA	4.42 (0.04)	4.21 (0.04)	7.14 (0.15)	< 0.001
WHtR	0.55 (0.00)	0.55 (0.00)	0.57 (0.02)	0.070
BMI	22.77 (0.16)	22.68 (0.17)	24.01 (0.45)	0.010
**Sex**				0.520
Female	1406 (30.09)	1305 (30.35)	101 (26.73)	
Male	3207 (69.91)	2968 (69.65)	239 (73.27)	
**Education**				0.020
Primary school	1146 (22.28)	1101 (22.97)	45 (13.28)	
High school or above	3467 (77.72)	3172 (77.03)	295 (86.72)	
**Marital**				0.030
Married	903 (16.70)	869 (17.30)	34 (8.92)	
Separated	306 (7.13)	293 (7.30)	13 (4.96)	
Never married	3404 (76.17)	3111 (75.40)	293 (86.12)	
**Diabetes**				0.010
No	3786 (81.87)	3542 (82.81)	244 (69.63)	
Yes	827 (18.13)	731 (17.19)	96 (30.37)	
**Hypertension**				0.120
No	2073 (42.71)	1953 (43.42)	120 (33.45)	
Yes	2540 (57.29)	2320 (56.58)	220 (66.55)	
**Hyperlipidemia**				0.002
No	2322 (48.97)	2208 (50.40)	114 (30.19)	
Yes	2291 (51.03)	2065 (49.60)	226 (69.81)	
**Healthy Lifestyle Score**				0.080
0	69 (1.90)	66 (2.00)	3 (0.60)	
1	618 (13.58)	552 (13.26)	66 (17.84)	
2	1568 (33.55)	1445 (32.73)	123 (44.36)	
3	1435 (30.44)	1354 (31.23)	81 (20.19)	
4	705 (16.18)	644 (16.22)	61 (15.64)	
5	218 (4.34)	212 (4.56)	6 (1.37)	

Percentages were adjusted for CHARLS survey weights. The P-value was calculated using a chi-square test and Students T test after considering the sampling weights. P-value < 0.05.

### 3.2 Multivariate logistic regression analysis of TyG, TyG-BMI, TyG-WHtR, TyG-WC with HUA

Among NHANES participants, we found statistically significant associations between different levels of TyG, TyG-BMI, TyG-WHtR and TyG-WC and HUA (*P-value* < 0.001). In Model 1, TyG [Q2, 1.88 (1.50, 2.34); Q3, 3.17 (2.63, 3.82); Q4, 4.92 (4.07, 5.95)], TyG-BMI [Q2, 2.66 (2.17, 3.24); Q3, 4.49 (3.69, 5.47). Q4, 9.06 (7.63, 10.75)], TyG-WHtR [Q2, 2.23 (1.82, 2.73); Q3, 3.96 (3.28, 4.78); Q4, 7.96 (6.65, 9.53)], TyG-WC [Q2, 2.66 (2.14, 3.29); Q3. 4.45 (3.67, 5.41); Q4, 9.98 (8.29, 12.03)] may be risk factors for HUA and may increase the risk of developing HUA. In Model 2, TyG [Q2, 1.77 (1.42, 2.22); Q3, 2.88 (2.38, 3.48); Q4, 4.31 (3.54, 5.25)], TyG-BMI [Q2, 2.34 (1.91, 2.86); Q3, 3.91 (3.21, 4.76)); Q4, 8.35 (7.04, 9.91)], TyG-WHtR (Q2, 2.12 (1.74, 2.57); Q3, 3.69 (3.07, 4.44); Q4, 7.96 (6.63, 9.54)), TyG-WC [Q2, 2.50 (2.03, 3.08); Q3, 4.06 (3.35, 4.92); Q4, 9.07 (7.53, 10.93)] may be risk factors for HUA and may increase the risk of developing HUA. In Model 3, TyG [Q2, 1.58 (1.26, 1.98); Q3, 2.36 (1.94, 2.88); Q4, 3.21 (2.61, 3.94)], TyG-BMI [Q2, 2.14 (1.74, 2.65) 3.38 (2.74, 4.17)); Q4, 6.67 (5.55, 8.02)], TyG-WHtR [Q2, 1.92 (1.56, 2.36); Q3, 3.14 (2.56, 3.85); Q4, 6.28 (5.12, 7.69)], TyG-WC [Q2, 2.32 (1.85, 2.90); Q3, 3.51 (2.84, 4.34); Q4, 7.32 (5.95, 9.02)] may be risk factors for HUA and may increase the risk of developing HUA. As shown in **Table 2-1**.

**Table 2-1:** Multivariate logistic regression results of the relationship between TyG, TyG BMI, TyG WHtR and TyG WC with HUA in NHANES, separately.

Parameters			Model 1		Model 2		Model 3	
			*P-value*	*OR (95%CI)*	*P-value*	*OR (95%CI)*	*P-value*	*OR (95%CI)*
TyG								
[5.65, 8.16]	Q1	N = 3569	Ref		Ref		Ref	
(8.16, 8.58]	Q2	N = 3556	< 0.001	1.88 (1.50, 2.34)	< 0.001	1.77 (1.42, 2.22)	< 0.001	1.58 (1.26, 1.98)
(8.58, 9.03]	Q3	N = 3563	< 0.001	3.17 (2.63, 3.82)	< 0.001	2.88 (2.38, 3.48)	< 0.001	2.36 (1.94, 2.88)
(9.03, 13.40]	Q4	N = 3571	< 0.001	4.92 (4.07, 5.95)	< 0.001	4.31 (3.54, 5.25)	< 0.001	3.21 (2.61, 3.94)
TyG BMI								
[116.43, 201.82]	Q1	N = 3565	ref		ref		ref	
(201.82, 238.85]	Q2	N = 3565	< 0.001	2.66 (2.17, 3.24)	< 0.001	2.34 (1.91, 2.86)	< 0.001	2.14 (1.74, 2.65)
(238.85, 282.22]	Q3	N = 3564	< 0.001	4.49 (3.69, 5.47)	< 0.001	3.91 (3.21, 4.76)	< 0.001	3.38 (2.74, 4.17)
(282.22, 600.55]	Q4	N = 3565	< 0.001	9.06 (7.63, 10.75)	< 0.001	8.35 (7.04, 9.91)	< 0.001	6.67 (5.55, 8.02)
TyG WHtR								
[2.58, 4.29]	Q1	N = 3564	ref		ref		ref	
(4.29, 4.97]	Q2	N = 3564	< 0.001	2.23 (1.82, 2.73)	< 0.001	2.12 (1.74, 2.57)	< 0.001	1.92 (1.56, 2.36)
(4.97, 5.67]	Q3	N = 3564	< 0.001	3.96 (3.28, 4.78)	< 0.001	3.69 (3.07, 4.44)	< 0.001	3.14 (2.56, 3.85)
(5.67, 10.33]	Q4	N = 3567	< 0.001	7.96 (6.65, 9.53)	< 0.001	7.96 (6.63, 9.54)	< 0.001	6.28 (5.12, 7.69)
TyG WC								
[431.53, 722.51]	Q1	N = 3565	ref		ref		ref	
(722.51, 837.16]	Q2	N = 3565	< 0.001	2.66 (2.14, 3.29)	< 0.001	2.50 (2.03, 3.08)	< 0.001	2.32 (1.85, 2.90)
(837.16, 956.17]	Q3	N = 3564	< 0.001	4.45 (3.67, 5.41)	< 0.001	4.06 (3.35, 4.92)	< 0.001	3.51 (2.84, 4.34)
(956.17, 1646.76]	Q4	N = 3565	< 0.001	9.98 (8.29, 12.03)	< 0.001	9.07 (7.53, 10.93)	< 0.001	7.32 (5.95, 9.02)

Model 1, No adjustment for any potential influence factors.

Model 2, Adjusted for Sex, Age.

Model 3, Adjusted for Sex, Age, Marital, Education levels, Hypertension, Diabetes, Hypertriglyceridemia and Healthy Lifestyle Score.

Among CHARLS participants, we found higher levels of TyG, TyG-BMI, TyG-WHtR and TyG-WC to be statistically significantly associated with HUA (*P-value* < 0.05). In Model1, TyG [Q4, 3.74 (1.84, 7.60)], TyG-BMI [Q3, 2.62 (1.12, 6.15); Q4, 4.05 (1.93, 8.49)], TyG-WHtR [Q3, 2.30 (1.04, 5.07); Q4, 3.83 (1.75, 8.37)], TyG-WC [Q3, 2.54 (1.11, 5.83); Q4, 4.19 (1.92, 9.15)] may be risk factors for HUA and may increase the risk of developing HUA. In Model2, TyG [Q4, 3.69 (1.84, 7.39)], TyG-BMI [Q3, 2.80 (1.20, 6.57); Q4, 4.68 (2.26, 9.69)], TyG-WHtR [Q3, 2.44 (1.13, 5.28); Q4, 4.05 (1.93, 8.52)], TyG-WC [Q3, 2.51 (1.11, 5.71); Q4, 3.92 (1.85, 8.27)] may be risk factors for HUA and may increase the risk of developing HUA. In Model3, TyG [Q4, 2.36 (1.08, 5.15)], TyG-BMI [Q3, 2.60 (1.05, 6.41); Q4, 3.70 (1.64, 8.32)], TyG-WHtR [Q4, 2.84 (1.23, 6.55)], TyG-WC [Q4, 2.85 (1.23, 6.59)] may be risk factors for HUA and may increase the risk of developing HUA. As shown in **Table 2-2**.

**Table 2-2:** Multivariate logistic regression results of the relationship between TyG, TyG BMI, TyG WHtR and TyG WC with HUA in CHARLS, separately.

Parameters			Model 1		Model 2		Model 3	
			P-value	OR (95%CI)	P-value	OR (95%CI)	P-value	OR (95%CI)
TyG								
[1.88, 2.11]	Q1	N= 1154	Ref		Ref		Ref	
(2.11, 2.16]	Q2	N= 1150	0.616	1.24 (0.53, 2.90)	0.569	1.28 (0.54, 3.03)	0.649	1.22 (0.52, 2.90)
(2.16, 2.20]	Q3	N= 1158	0.286	1.54 (0.70, 3.39)	0.305	1.52 (0.68, 3.38)	0.709	1.17 (0.51, 2.70)
(2.20, 2.48]	Q4	N= 1151	< 0.001	3.74 (1.84, 7.60)	< 0.001	3.69 (1.84, 7.39)	0.031	2.36 (1.08, 5.15)
TyG BMI								
[13.69, 170.72]	Q1	N= 1149	ref		ref		ref	
(170.72, 193.59]	Q2	N= 1157	0.262	1.61 (0.70, 3.67)	0.175	1.78 (0.77, 4.11)	0.157	1.91 (0.78, 4.66)
(193.59, 221.21]	Q3	N= 1156	0.027	2.62 (1.12, 6.15)	0.018	2.80 (1.20, 6.57)	0.039	2.60 (1.05, 6.41)
(221.21, 433.19]	Q4	N= 1151	< 0.001	4.05 (1.93, 8.49)	<0.0001	4.68 (2.26, 9.69)	0.002	3.70 (1.64, 8.32)
TyG WHtR								
[0.60, 4.17]	Q1	N= 1156	ref		ref		ref	
(4.17, 4.75]	Q2	N= 1143	0.729	1.16 (0.50, 2.70)	0.672	1.20 (0.52, 2.76)	0.829	1.10 (0.47, 2.54)
(4.75, 5.32]	Q3	N= 1159	0.04	2.30 (1.04, 5.07)	0.023	2.44 (1.13, 5.28)	0.066	2.13 (0.95, 4.75)
(5.32, 7.49]	Q4	N= 1155	< 0.001	3.83 (1.75, 8.37)	< 0.001	4.05 (1.93, 8.52)	0.014	2.84 (1.23, 6.55)
TyG WC								
[95.58, 646.62]	Q1	N= 1154	ref		ref		ref	
(646.62, 726.27]	Q2	N= 1151	0.261	1.60 (0.70, 3.65)	0.296	1.55 (0.68, 3.50)	0.365	1.47 (0.64, 3.41)
(726.27, 811.38]	Q3	N= 1157	0.028	2.54 (1.11, 5.83)	0.028	2.51 (1.11, 5.71)	0.081	2.20 (0.91, 5.34)
(811.38, 1155.16]	Q4	N= 1151	< 0.001	4.19 (1.92, 9.15)	< 0.001	3.92 (1.85, 8.27)	0.015	2.85 (1.23, 6.59)

Model 1, No adjustment for any potential influence factors.

Model 2, Adjusted for Sex, Age.

Model 3, Adjusted for Sex, Age, Marital, Education levels, Hypertension, Diabetes, Hypertriglyceridemia and Healthy Lifestyle Score.

### 3.3 Sex stratified analysis

Among NHANES participants of different sex, we found statistically significant associations between higher levels of TyG, TyG-BMI, TyG-WHtR, and TyG-WC and HUA (p value < 0.05). TyG [Male (Q2, 1.58 (1.20, 2.08); Q3, 2.26 (1.74, 2.92); Q4, 2.72 (2.06, 3.57)), Female (Q2, 1.44 (1.06, 1.94);Q3, 2.17 (1.65, 2.85);Q4, 3.65 (2.74, 4.85))], TyG BMI [Male (Q2, 2.02 (1.52, 2.69); Q3, 3.26 (2.45, 4.35); Q4, 5.83 (4.52, 7.53)), Female (Q2, 2.25 (1.60, 3.18); Q3, 3.21 (2.30, 4.49); Q4, 8.09 (6.01, 10.88))], TyG WHtR [Male (Q2, 1.72 (1.35, 2.20); Q3, 2.90 (2.23, 3.78); Q4, 4.94 (3.84, 6.36)), Female (Q2, 2.46 (1.63, 3.70); Q3, 3.78 (2.51, 5.68); Q4, 9.82 (6.75, 14.27))], TyG WC [Male (Q2, 2.28 (1.67, 3.12); Q3, 3.08 (2.29, 4.13); Q4, 6.50 (4.90, 8.63)), Female (Q2, 2.07 (1.52, 2.82); Q3, 3.73 (2.70, 5.13); Q4, 8.31 (6.17, 11.19))]. As shown in **Table 3-1**.

**Table 3-1:** Multivariate logistic regression results of the relationship between TyG, TyG BMI, TyG WHtR and TyG WC with HUA in different sex in NHANES, separately.

Parameters	Q1	Q2		Q3		Q4	
		*P-value*	*OR (95%CI)*	*P-value*	*OR (95%CI)*	*P-value*	*OR (95%CI)*
TyG							
Male	ref	0.001	1.58 (1.20, 2.08)	< 0.001	2.26 (1.74, 2.92)	< 0.001	2.72 (2.06, 3.57)
Female	ref	0.02	1.44 (1.06, 1.94)	< 0.001	2.17 (1.65, 2.85)	< 0.001	3.65 (2.74, 4.85)
TyG BMI							
Male	ref	< 0.001	2.02 (1.52, 2.69)	< 0.001	3.26 (2.45, 4.35)	< 0.001	5.83 (4.52, 7.53)
Female	ref	< 0.001	2.25 (1.60, 3.18)	< 0.001	3.21 (2.30, 4.49)	< 0.001	8.09 (6.01, 10.88)
TyG WHtR							
Male	ref	< 0.001	1.72 (1.35, 2.20)	< 0.001	2.90 (2.23, 3.78)	< 0.001	4.94 (3.84, 6.36)
Female	ref	< 0.001	2.46 (1.63, 3.70)	< 0.001	3.78 (2.51, 5.68)	< 0.001	9.82 (6.75, 14.27)
TyG WC							
Male	ref	< 0.001	2.28 (1.67, 3.12)	< 0.001	3.08 (2.29, 4.13)	< 0.001	6.50 (4.90, 8.63)
Female	ref	< 0.001	2.07 (1.52, 2.82)	< 0.001	3.73 (2.70, 5.13)	< 0.001	8.31 (6.17, 11.19)

Model, Adjusted for Age, Marital, Education levels, Hypertension, Diabetes, Hypertriglyceridemia and Healthy Lifestyle Score.

Among CHARLS participants of different sex, we found statistically significant associations between higher levels of TyG, TyG-BMI, TyG-WHtR, and TyG-WC and HUA (p value < 0.05). TyG [Female (Q4, 4.84 (1.77, 13.23))], TyG BMI [Female (Q2, 4.40 (1.38, 14.04); Q3, 4.88 (1.42, 16.78); Q4, 8.16 (2.79, 23.89))], TyG WHtR [Female (Q3, 3.49 (1.15, 10.64); Q4, 5.55 (1.99, 15.47))], TyG WC [Female (Q4, 5.27 (2.10, 13.22))]. As shown in **Table 3-2**.

**Table 3-2:** Multivariate logistic regression results of the relationship between TyG, TyG BMI, TyG WHtR and TyG WC with HUA in different sex in Charls, separately.

Parameters	Q1	Q2		Q3		Q4	
		P-value	OR (95%CI)	P-value	OR (95%CI)	P-value	OR (95%CI)
TyG							
Male	ref	0.46	0.60 (0.16, 2.29)	0.44	0.57 (0.14, 2.37)	0.31	0.50 (0.13, 1.90)
Female	ref	0.29	1.83 (0.60, 5.63)	0.35	1.67 (0.56, 4.93)	0.002	4.84 (1.77, 13.23)
TyG BMI							
Male	ref	0.45	0.58 (0.14, 2.36)	0.94	0.95 (0.28, 3.20)	0.88	0.89 (0.19, 4.24)
Female	ref	0.01	4.40 (1.38, 14.04)	0.01	4.88 (1.42, 16.78)	< 0.001	8.16 (2.79, 23.89)
TyG WHtR							
Male	ref	0.38	0.55 (0.14, 2.14)	0.46	1.70 (0.42, 6.80)	0.68	0.66 (0.09, 5.06)
Female	ref	0.16	2.27 (0.73, 7.12)	0.03	3.49 (1.15, 10.64)	0.001	5.55 (1.99, 15.47)
TyG WC							
Male	ref	0.79	0.82 (0.19, 3.51)	0.64	1.40 (0.34, 5.70)	0.37	0.46 (0.08, 2.51)
Female	ref	0.15	2.05 (0.76, 5.49)	0.07	2.85 (0.92, 8.77)	< 0.001	5.27 (2.10, 13.22)

Model, Adjusted for Age, Marital, Education levels, Hypertension, Diabetes, Hypertriglyceridemia and Healthy Lifestyle Score.

### 3.4 RCS analysis

The RCS was used to determine if there was a nonlinear association between TyG, TyG-BMI, TyG-WHtR, TyG-WC, and HUA. Among NHANES participants, results showed a nonlinear association between TyG, TyG-BMI, TyG-WHtR, TyG-WC, and HUA (*P-value* < 0.05). As shown in **Figure 2-1**. Among CHARLS participants, the results showed a nonlinear association between TyG, TyG-BMI, TyG-WHtR, TyG-WC, and HUA (*P-value* < 0.05). As shown in **Figure 2-2**.

**Figure 2-1:**
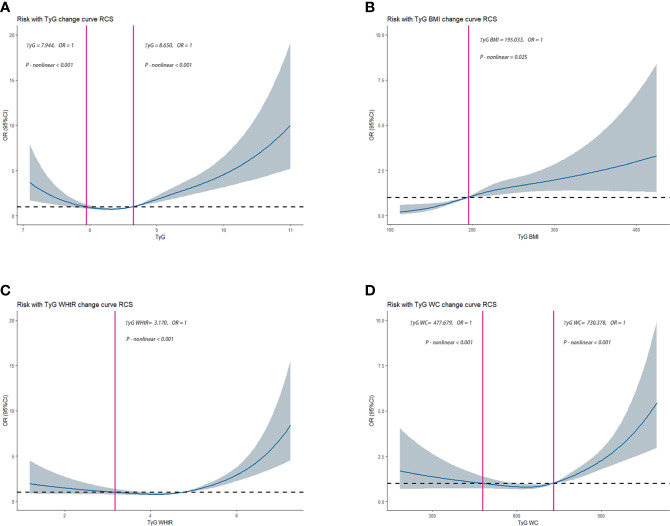
Dose–response relationships between TyG, TyG-BMI, TyG-WHtR, TyG-WC, and HUA in NHANES, separately **(A)**, TyG **(B)**, TyG-BMI **(C)**, TyG-WHtR **(D)**, TyG-WC. OR (95% CI) (shaded areas) were adjusted for Sex, Age, Marital, Education levels, Hypertension, Diabetes, Hypertriglyceridemia and Healthy Lifestyle Score. Vertical red solid lines indicate the minimal threshold for the beneficial association with estimated OR = 1. OR, odds ratio.

**Figure 2-2:**
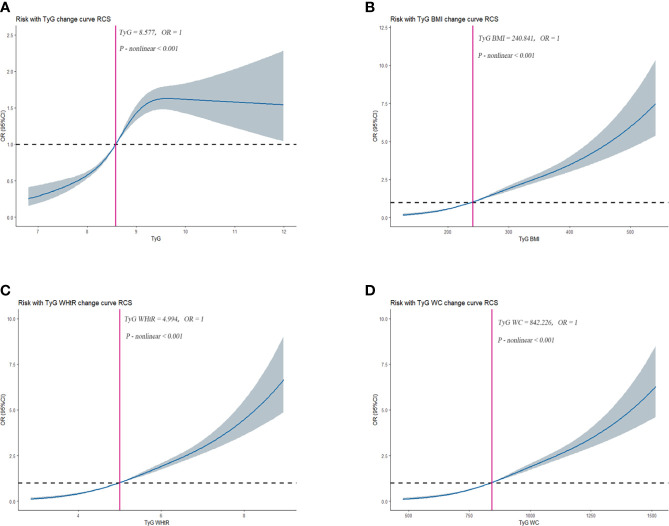
Dose–response relationships between TyG, TyG-BMI, TyG-WHtR, TyG-WC, and HUA in CHARLS, separately **(A)**, TyG **(B)**, TyG-BMI **(C)**, TyG-WHtR **(D)**, TyG-WC. OR (95% CI) (shaded areas) were adjusted for Sex, Age, Marital, Education levels, Hypertension, Diabetes, Hypertriglyceridemia and Healthy Lifestyle Score. Vertical red solid lines indicate the minimal threshold for the beneficial association with estimated OR = 1. OR, odds ratio.

### 3.5 Sensitivity analysis

We found that different levels of TyG, TyG-BMI, TyG-WHtR and TyG-WC were still statistically significantly associated with HUA (*P-value* < 0.05) in NHANES participants who did not have hypertension, hyperlipidemia, and diabetes. In Model 1, TyG [Q4, 3.00 (1.70, 5.29)], TyG-BMI [Q3, 3.53 (1.96, 6.38); Q4, 6.61 (3.71, 11.78)], TyG-WHtR [Q3, 2.82 (1.47, 5.41); Q4, 5.86 (3.14, 10.93)], TyG-WC [Q3, 3.26 (1.72, 6.17); Q4, 6.40 (3.38, 12.13)] may be risk factors for HUA and may increase the risk of developing HUA. In Model 2, TyG [Q4, 2.40 (1.36, 4.23)], TyG-BMI [Q3, 2.97 (1.63, 5.40); Q4, 5.78 (3.24, 10.32)], TyG-WHtR [Q3, 2.99 (1.52, 5.91); Q4, 6.35 (3.29, 12.23)], TyG-WC [Q3, 2.83 (1.48, 5.39); Q4, 5.36 (2.84, 10.12)] may be risk factors for HUA and may increase the risk of developing HUA.

In Model3, TyG [Q4, 2.16 (1.20, 3.88)], TyG-BMI [Q3, 3.04 (1.64, 5.63); Q4, 5.95 (3.22, 11.02)], TyG-WHtR [Q3, 3.07 (1.55, 6.08); Q4, 6.54 (3.38, 12.66)], and TyG-WC (Q3, 2.85 (1.52, 5.36); Q4, 5.35 (2.85, 10.01)) may be risk factors for HUA and may increase the risk of developing HUA. As shown in **Table 4-1**.

**Table 4-1:** Multivariate logistic regression results of the relationship between TyG, TyG BMI, TyG WHtR and TyG WC with HUA in NHANES, separately. Sensitivity analyses excluding hypertension, hyperlipidemia, and diabetes.

Parameters			Model 1		Model 2		Model 3	
			*P-value*	*OR (95%CI)*	*P-value*	*OR (95%CI)*	*P-value*	*OR (95%CI)*
TyG								
[6.19, 7.79]	Q1	N= 746	ref		ref		ref	
(7.79, 8.08]	Q2	N= 744	0.476	0.80 (0.44, 1.47)	0.361	0.76 (0.41, 1.38)	0.292	0.72 (0.39, 1.33)
(8.08, 8.39]	Q3	N= 740	0.356	1.29 (0.75, 2.22)	0.544	1.18 (0.68, 2.05)	0.666	1.13 (0.65, 1.96)
(8.39, 10.24]	Q4	N= 750	< 0.001	3.00 (1.70, 5.29)	0.003	2.40 (1.36, 4.23)	0.01	2.16 (1.20, 3.88)
TyG BMI								
[116.43, 173.08]	Q1	N= 745	ref		ref		ref	
(173.08, 197.31]	Q2	N= 745	0.255	1.48 (0.75, 2.94)	0.368	1.37 (0.69, 2.76)	0.371	1.37 (0.68, 2.75)
(197.31, 230.84]	Q3	N= 745	< 0.001	3.53 (1.96, 6.38)	< 0.001	2.97 (1.63, 5.40)	< 0.001	3.04 (1.64, 5.63)
(230.84, 497.55]	Q4	N= 745	< 0.001	6.61 (3.71, 11.78)	< 0.001	5.78 (3.24, 10.32)	< 0.001	5.95 (3.22, 11.02)
TyG WHtR								
[2.70, 3.67]	Q1	N= 746	ref		ref		ref	
(3.67, 4.09]	Q2	N= 745	0.211	1.56 (0.78, 3.13)	0.193	1.62 (0.78, 3.34)	0.183	1.63 (0.79, 3.37)
(4.09, 4.66]	Q3	N= 743	0.002	2.82 (1.47, 5.41)	0.002	2.99 (1.52, 5.91)	0.001	3.07 (1.55, 6.08)
(4.66, 8.29]	Q4	N= 746	< 0.001	5.86 (3.14, 10.93)	< 0.001	6.35 (3.29, 12.23)	< 0.001	6.54 (3.38, 12.66)
TyG WC								
[431.53, 623.05]	Q1	N= 745	ref		ref		ref	
(623.05, 695.48]	Q2	N= 745	0.339	1.41 (0.69, 2.89)	0.502	1.28 (0.62, 2.62)	0.54	1.25 (0.61, 2.56)
(695.48, 792.20]	Q3	N= 745	< 0.001	3.26 (1.72, 6.17)	0.002	2.83 (1.48, 5.39)	0.001	2.85 (1.52, 5.36)
(792.20, 1454.51]	Q4	N= 745	< 0.001	6.40 (3.38, 12.13)	< 0.001	5.36 (2.84, 10.12)	< 0.001	5.35 (2.85, 10.01)

Model 1, No adjustment for any potential influence factors.

Model 2, Adjusted for Sex, Age.

Model 3, Adjusted for Sex, Age, Marital, Education levels and Healthy Lifestyle Score.

We found that TyG remained statistically significantly associated with HUA in CHARLS participants without hypertension, hyperlipidemia, and diabetes (*P-value* < 0.05), and did not find statistically significant associations of TyG-BMI, TyG-WHtR and TyG-WC with HUA. In Model1, 2, and 3, TyG [Q3, 0.001(0.001,0.001)]. As shown in **Table 4-2**.

**Table 4-2:** Multivariate logistic regression results of the relationship between TyG, TyG BMI, TyG WHtR and TyG WC with HUA in CHARLS, separately. Sensitivity analyses excluding hypertension, hyperlipidemia, and diabetes.

Parameters			Model 1		Model 2		Model 3	
			P-value	OR (95%CI)	P-value	OR (95%CI)	P-value	OR (95%CI)
TyG								
[6.19, 7.79]	Q1	N= 746	ref		ref		ref	
(7.79, 8.08]	Q2	N= 744	0.223	0.24 (0.03, 2.38)	0.143	0.19 (0.02, 1.75)	0.113	0.14 (0.01, 1.60)
(8.08, 8.39]	Q3	N= 740	<0.0001	0.01 (0.01, 0.01)	<0.0001	0.01 (0.01, 0.01)	<0.0001	0.01 (0.01, 0.01)
(8.39, 10.24]	Q4	N= 750	0.229	0.37 (0.07, 1.90)	0.114	0.29 (0.06, 1.35)	0.088	0.24 (0.05, 1.23)
TyG BMI								
[116.43, 173.08]	Q1	N= 745	ref		ref		ref	
(173.08, 197.31]	Q2	N= 745	0.339	0.36 (0.04, 2.94)	0.346	0.35 (0.04, 3.19)	0.594	0.54 (0.06, 5.18)
(197.31, 230.84]	Q3	N= 745	0.92	0.91 (0.13, 6.28)	0.85	1.21 (0.17, 8.42)	0.558	1.91 (0.22, 16.65)
(230.84, 497.55]	Q4	N= 745	0.483	0.41 (0.03, 4.95)	0.896	0.84 (0.06, 11.79)	0.802	1.46 (0.08, 27.73)
TyG WHtR								
[2.70, 3.672	Q1	N= 746	ref		ref		ref	
(3.67, 4.09]	Q2	N= 745	0.532	2.05 (0.21, 19.86)	0.576	2.10 (0.16, 28.32)	0.509	2.30 (0.19, 27.57)
(4.09, 4.66]	Q3	N= 743	0.843	0.78 (0.07, 9.05)	0.927	0.89 (0.08, 10.60)	0.956	0.93 (0.08, 11.61)
(4.66, 8.29]	Q4	N= 746	0.715	0.59 (0.04, 9.92)	0.678	0.59 (0.05, 7.35)	0.917	0.88 (0.07, 10.99)
TyG WC								
[431.53, 623.05]	Q1	N= 745	ref		ref		ref	
(623.05, 695.48]	Q2	N= 745	0.667	1.63 (0.17, 15.33)	0.664	1.72 (0.15, 20.11)	0.675	1.72 (0.13, 22.03)
(695.48, 792.20]	Q3	N= 745	0.923	1.13 (0.10, 13.48)	0.985	0.98 (0.07, 12.82)	0.938	1.11 (0.08, 15.33)
(792.20, 1454.51]	Q4	N= 745	0.718	0.60 (0.04, 10.00)	0.764	0.67 (0.05, 9.51)	0.955	0.92 (0.06, 14.69)

Model 1, No adjustment for any potential influence factors.

Model 2, Adjusted for Sex, Age.

Model 3, Adjusted for Sex, Age, Marital, Education levels and Healthy Lifestyle Score.

## 4 Discussion

The study presents a novel discovery based on consistent findings from the Chinese CHARLS and the US NHANES databases. The research identifies TyG, TyG-BMI, TyG-WHtR, and TyG-WC as potential risk factors for HUA in the general populations of China and the United States, indicating a heightened risk of developing HUA. These indicators promote that the risk of developing HUA is equally increased in men and women. Furthermore, TyG and its derived metrics only have an effect on HUA at higher levels. Notably, this study is the first to incorporate healthy lifestyle factors, sampling weights, and combined data from Chinese and U.S. general populations in the analysis of TyG and its derivatives concerning the risk of HUA.

Previous studies have demonstrated a close relationship between HUA and IR, and the gold standard for IR assessment is the Hyperinsulinemic-euglycemic clamp (36). However, the Hyperinsulinemic-euglycemic clamp (HEC) is challenging to implement in clinical settings because of its invasive, complex, and time-intensive procedures. Alternatively, the Homeostatic Model Assessment of Insulin Resistance (HOMA-IR) indices offer a simpler approach for assessing IR (36). TyG was proposed by Simal-Mendia et al. (37) as a simple, practical and usable alternative marker to identify IR. It has been shown that TyG maintains good agreement with HEC and HOMA-IR (13). In clinical practice, TyG provides additional options for evaluating IR and reducing the risk of IR-related diseases. This new biomarker is recognized as a valuable tool for diagnosing various chronic conditions. Previous research has demonstrated that TyG markers are linked to conditions such as diabetes, hypertension, metabolic syndrome, atherosclerosis, and the long-term risk of cardiovascular disease (38, 39). Due to its ease of use and cost-effectiveness, the TyG index has been utilized as a proxy for IR in extensive epidemiological studies. It has demonstrated significant associations with TyG-BMI, TyG-WC, and TyG-WTHR (14, 40). Previous studies have demonstrated that obesity markers (BMI, WC and WHTR) are associated with HUA (41), while the release of pro-inflammatory cytokines from adipose tissue can exacerbate IR (42, 43). The superiority of combining TyG with obesity indicators has been demonstrated in a student-oriented study, stating that TyG and its derivatives may increase the prevalence risk of HUA in specific groups (41). TyG and TyG-BMI indices were used as IR biomarkers in a cross-sectional study among Chinese older adults, and these markers showed significant associations with increased risk of HUA or hypertension, alone or in combination (44), which is similar to and supports our findings.

Physiological concentrations of SUA, a metabolic byproduct of purine nucleotides, have beneficial antioxidant effects and act as free radical scavengers (45). SUA also provides protective effects such as anti-DNA damage, anti-aging effects, and delayed cognitive decline. However, high concentrations of uric acid can lead to HUA and the aggregation of reactive oxygen species (ROS) under stressful conditions like hypoxia and ischemia, contributing to oxidative stress (45). Increased ROS formation resulting from HUA can decrease the transcription factors necessary for insulin gene expression, leading to reduced insulin synthesis and release (46). Elevated levels of SUA are associated with oxidative stress, which can impair glucose metabolism, decrease insulin sensitivity, and contribute to IR by upregulating insulin receptor substrate 1 phosphorylation and increasing ROS production (47–49). IR promotes SUA synthesis through the hexose monophosphate pathway and reduces SUA renal excretion (48, 49). Additionally, insulin enhances renal reabsorption of uric acid by activating glucose transporter protein (Glut 9) and other urate transporter proteins, ultimately leading to elevated uric acid levels (50). These results suggest that TyG and its derivatives reflect metabolic abnormalities and suggest the possibility that participants are at increased risk of developing HUA. Additionally, NHANES results were that TyG, TyG-BMI, TyG-WHtR, and TyG-WC were able to increase the risk of the disease in both men and women, and Charls results were that TyG and its derived indices were better predictors of the risk of HUA in women. TyG and its derived indices were positively associated with HUA (22). And it can be considered a good indicator for screening for HUA regardless of the participant’s obesity status (51). Previous studies have noted that the predictive ability of both TyG and its derived indices for HU risk is better in women than in men (52).This is similar to our findings. This study, conducted in the United States and China, utilized a larger population sample to represent the general population. It considered population weighting and healthy lifestyles, revealing an association between TyG and its derivatives with HUA. These findings could help shape future programs aimed at preventing HUA. However, the cross-sectional design of the study limits the ability to establish a causal relationship between IR and HUA. A cohort study suggested that a high TyG index was associated with an increased risk of CKD (18), and that renal function (including GFR) was a risk factor for HUA (21).

Although our findings suggest a relationship between TyG and HUA, the specific mechanism remains unclear. The most common mechanism is associated with IR, with compensatory hyperinsulinemia following IR, resulting in reduced uric acid excretion through renal tubule sodium reabsorption, leading to HUA (53). However, epidemiological evidence needs to be validated with longer follow-up and larger samples. It is important to note the low representation of adults aged 20-44 years in the Chinese CHARLS study, which was dominated by participants aged 45 years and older. Despite this, participants aged 20-44 years were included in the Chinese CHARLS study to maintain consistency with the NHANES study. Despite controlling for various covariates in the analyses, the original design of the CHARLS study may still have uncollected confounders, such as dietary factors like meat, vegetables, and dairy intake. Healthy lifestyles were calculated in NHANES, considering the HEI. The result of the study was a significant association between TyG and its derived indices and hyperuricemia (HUA). We cannot exclude unmeasured influences, such as lack of information on dietary habits, genetic predisposition, etc., that may have influenced the results. Sensitivity analysis showed that the NHANES study results were consistent with the main analysis, indicating that TyG and its derived indicators may be risk factors for HUA. However, results from CHARLS studies are inconsistent, possibly due to residual effects, unmeasured confounders, and measurement error that influence the analysis. Nonetheless, the main results of the NHANES and CHARLS studies are consistent. Future studies should focus on the critical values of TyG, TyG-BMI, TyG-WHtR and TyG-WC that affect HUA. This will facilitate the development of reliable, sensitive and convenient assessment tools for clinical research, epidemiological surveys and primary disease screening. Because we did not include clinical treatment measures in our study, it is not possible to speculate further on potential screening strategies or treatments for HUA. Our study implies that maintaining a low level of TyG index may be beneficial in preventing or mitigating the risk of developing HUA. This study has several limitations. First, this is a cross-sectional study, so only correlations, not causation and temporal associations, can be inferred. The cross-sectional design of this study limits the ability to determine a causal relationship between IR levels and HUA. To establish causality, longer follow-up investigations are needed. Additionally, we did not report the status of eGFR and failed to correct for it in the model. In this study we were unable to obtain complete measures of renal function, medical information on diuretics (and more in general CV drug therapies), information on renal progression or need for renal replacement therapy, longitudinal information on lipid profiles, race factors and genetic susceptibility, which may have an impact on the assessment of serum uric acid. In addition, although various covariates were controlled for in the analysis, there may still be confounding factors that were not captured by the original design of the CHARLS study, such as dietary habits, physical activity, and genetic predispositions. Finally, NHANES takes HEI into account when calculating healthy lifestyles. Information on diet, exercise, smoking, drinking, etc. is obtained through self-report, which may not be accurate enough.

## 5 Conclusion

The results showed that TyG, TyG-BMI, TyG-WHtR and TyG-WC were associated with a higher risk of HUA in Chinese and American populations. The predictive ability of each indicator for the risk of developing HUA was stronger in women than in men. Despite the limitations of this study, TyG has the potential to replace IR in assessing the prevalence of HUA. This study provides a reliable, sensitive and convenient assessment tool for the prevention of HUA.

## Data availability statement

Publicly available datasets were analyzed in this study. This data can be found here: CHARLS (http://charls.pku.edu.cn/pages/data/2011-charls-wave1/en.html) and NHANES (http://www.cdc.go/nchs/nhanes.htm).

## Ethics statement

The surveys were approved by the NCHS Research Ethics Review Board (Protocol #2011-17) in NHANES. All methods were performed in accordance with the relevant guidelines and regulations (Declaration of Helsinki). Informed consent was obtained from all subjects and/or their legal guardian(s). The studies involving human participants were reviewed and approved by the Ethics Review Committee of Peking University in CHARLS. The patients/participants provided their written informed consent to participate in this study.

## Author contributions

RG: Conceptualization, Formal analysis, Methodology, Supervision, Writing – original draft, Writing – review & editing. DD: Methodology, Supervision, Writing – original draft, Writing – review & editing. MT: Supervision, Writing – review & editing. XC: Supervision, Writing – review & editing. YZ: Supervision, Writing – review & editing. XM: Supervision, Writing – review & editing. GL: Funding acquisition, Supervision, Writing – original draft, Writing – review & editing.
